# Targeting ERβ in Macrophage Reduces Crown-like Structures in Adipose Tissue by Inhibiting Osteopontin and HIF-1α

**DOI:** 10.1038/s41598-019-52265-8

**Published:** 2019-10-31

**Authors:** Li Wang, Rui-peng Zhao, Xiao-yu Song, Wan-fu Wu

**Affiliations:** 1 Center for Nuclear Receptors and Cell Signaling, University of Houston, Houston, TX 77204 USA; 2 Department of Thyroid and Breast Surgery, The Affiliated Huaian No.1 People’s Hospital of Nanjing Medical University, 1 West Huanghe Road, Huaian, 223300 Jiangsu China

**Keywords:** Endocrine system and metabolic diseases, Cancer

## Abstract

Proinflammatory processes in adipose tissue contribute to development of breast cancer and insulin resistance. Crown-like structures (CLS) are histologic hallmarks of the proinflammatory process in adipose tissue. CLS are microscopic foci of dying adipocytes surrounded by macrophages mostly derived from monocytes in blood. Estrogen receptor β (ERβ) is expressed in microglia, macrophages within the central nervous system (CNS), where it evokes an anti-inflammatory response. The present study investigates the function of ERβ in macrophages within CLS. We report that even though monocytes in the blood have no detectable levels of ERβ, macrophages in CLS do express ERβ. In ERβ−/− mice, there was a significant increase in the number of CLS in both subcutaneous adipose tissue (SAT) and visceral adipose tissue (VAT). CLS in these mice were dominated by pro-inflammatory macrophages (M1 macrophages) with higher expression of osteopontin (OPN) and an increase in number of proliferating macrophages. In mice made obese by Western diet, treatment with an ERβ selective agonist (LY3201) reduced the number of CLS in both SAT and VAT with downregulation of OPN, activated hypoxia-inducible factor-1α (HIF-1α), proliferation and upregulation prolyl hydroxylase 2 (PHD2), the enzyme which prevents activation of HIF1α, in macrophages. We conclude that ERβ expression is induced in macrophages in CLS within adipose tissue where it plays a pivotal role in suppression of CLS. Thus ERβ agonists may be used to alleviate CLS-related breast cancer and insulin resistance in adipose tissue.

## Introduction

Crown-like structures (CLS), hallmarks of proinflammatory process in adipose tissue, are macrophages mostly derived from monocytes in blood surrounding dead or dying adipocytes. Adipose tissue proinflammatory processes are related to development of breast cancer^1–3^ and insulin resistance^4–6^. It has been reported that in inflamed breast white adipose tissue (WAT) both expression and activity of aromatase, the enzyme converting androgens to estrogens, are significantly increased^7^. Furthermore, there was an increase in the estrone/androstenedione ratio in breast tissue that contained CLS from postmenopausal breast cancer patients^8^. Macrophages in CLS are able to cause hyperinsulinemia and insulin resistance by releasing proinflammatory factors and free fatty acids^9,10^. CLS are also related to activation of NF-kB, expression of inducible nitric oxide synthase (iNOS, a driver of inflammation) and secretion of inflammatory factors, such as IL1β, IL6 and TNFα^11,12^. Inflamed adipose tissue can release cell-free DNA to stimulate insulin resistance^13,14^.

Osteopontin (OPN), a secreted glycoprotein, plays key roles in lots of physiological and pathological processes, including inflammation^15^. OPN expressed in activated macrophages plays a pivotal role in cell-mediated immunity^16,17^. OPN has also been reported to involve in macrophage infiltration and insulin resistance in obese mice^18^. It has been shown that OPN promotes survival and inhibits human monocytes apoptosis. Remarkably, the ability of OPN enhancing macrophage proliferation is similar to that of macrophage colony stimulating factor (M-CSF) in well differentiated macrophages^19^.

Hypoxia-inducible factor-1α (HIF-1α) is a transcription factor playing pivotal role in cellular adaptation to hypoxia and is tightly regulated by the oxygen tension^20,21^. HIF-1α hydroxylated by prolyl hydroxylases (PHD) is ubiquitinated and degraded under normoxia^22^. Under hypoxia, HIF-1α can be stabilized and translocated into nucleus, where it dimerizes with HIF-1β, the other subunit of HIF-1, and activates the gene transcription involving in survival in hypoxia. As obesity develops, HIF-1α is activated in macrophages in adipose tissue^23^. HIF-1α-activated macrophages are accumulated in CLS^24^. In CLS M1 polarization of macrophages is induced by HIF-1α activation through elevating glycolysis^25^.

Estrogen receptor β (ERβ) is expressed in microglia, macrophages within the central nervous system (CNS)^26,27^. In experimental autoimmune encephalomyelitis (EAE) mice stimulating ERβ evokes an anti-inflammatory response by inhibiting activated microglia^27,28^.

In the present study, by using ERβ knock out (ERβ−/−) mice and wild type (WT) mice with obesity induced by consumption of a Western diet (WD), we found that ERβ regulates the number of CLS in subcutaneous adipose tissue (SAT) and visceral adipose tissue (VAT) as well as activation of macrophages in CLS.

## Results

### ERβ is expressed in macrophages in CLS but not in monocytes in blood

**Figure 1:**
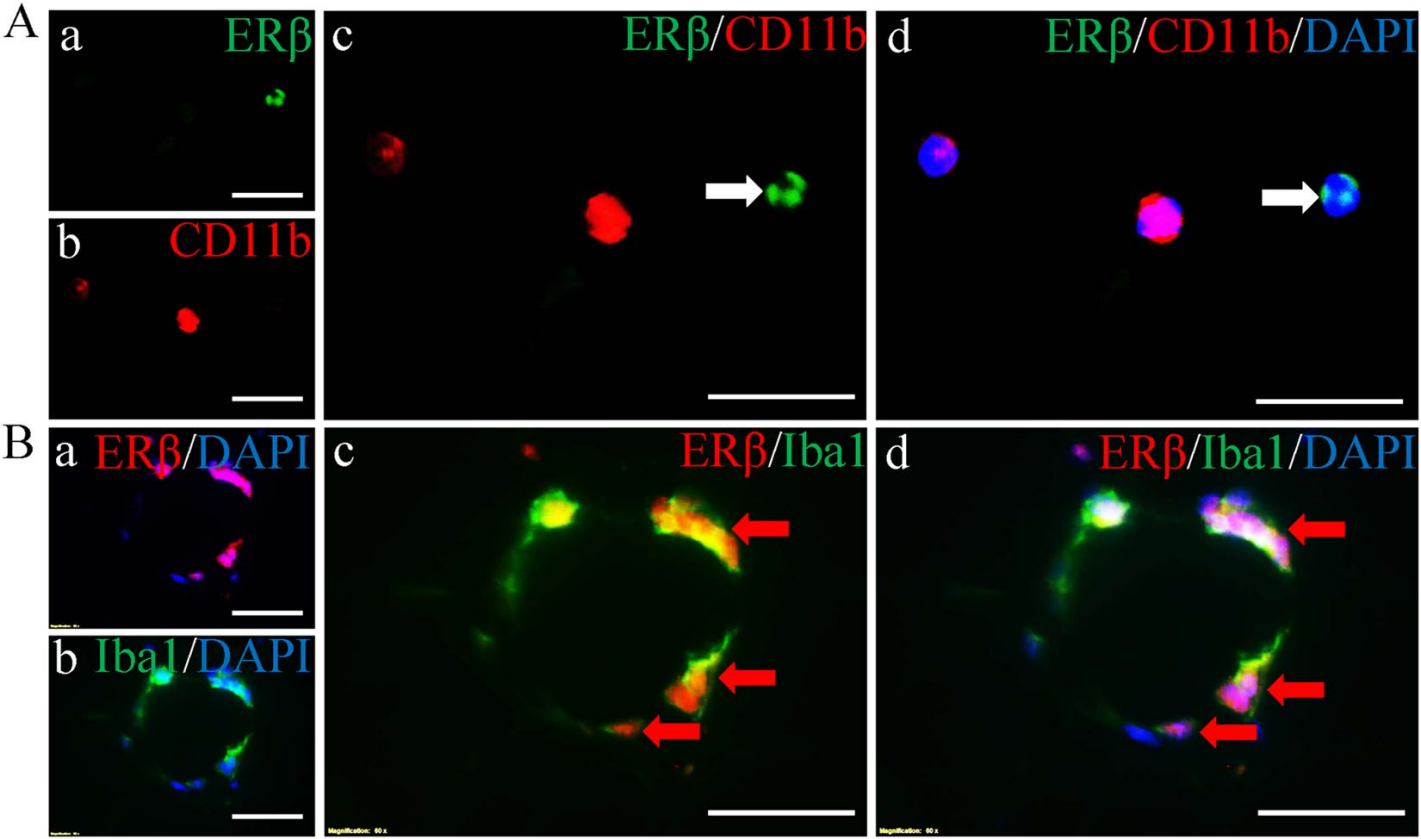
Expression of ERβ in monocytes of blood smear and macrophages in CLS. (**A**) ERβ and CD11b double fluorescent staining. There was no co-localization between ERβ (green) and CD11b (red) (white arrow) (c). Co-localization of ERβ (green), CD11b (red) and DAPI (blue) (white arrow) (d). (**B**) ERβ and Iba1 double fluorescent staining. There was co-localization between ERβ (red) and Iba1 (green) (c,d). The red arrows show ERβ and Iba1 co-localization in macrophages. Nuclei were counterstained with DAPI (blue). (Scale bars in **A,B**, 30 μm).

Macrophages in CLS are mostly derived from monocytes in blood. To identify whether ERβ is expressed in monocytes in blood or in macrophages in CLS, we used double fluorescent staining with antibodies for ERβ 503/CD11b (marker for mouse monocytes) in mouse blood smear samples and ERβ 503/Iba1 (maker for macrophages) on mouse adipose tissue. There was no co-localization between ERβ and CD11b although there were ERβ-nuclear-positive cells (Fig. 1Aa–d). However, ERβ co-localized in cells with Iba1 in CLS (Fig. 1Ba–d). Thus ERβ expresses in macrophages in CLS, not in monocytes. HeLa cells transfected with vehicle, ERβ1, ERβ2 or ERα were used to verify ERβ 503 antibody. The ERβ 503 antibody only stained HeLa cells transfected ERβ1 (Fig. s1Ab). No positive staining was found in HeLa cells transfected with vehicle, ERβ2 or ERα (Fig. s1Aa,c,d). To further confirm ERβ 503 antibody staining is specific on mouse tissue, immunohistochemistry was performed on SAT (from mammary glands) sections. ERβ was localized in the nuclei of the macrophages within CLS in SAT of WT mice. No ERβ positive cells were found in ERβ−/− mice (Fig. s1Ba,b). These results proved that the ERβ 503 antibody is specific.

### Loss of ERβ leads to an increase of CLS in both SAT and VAT

**Figure 2:**
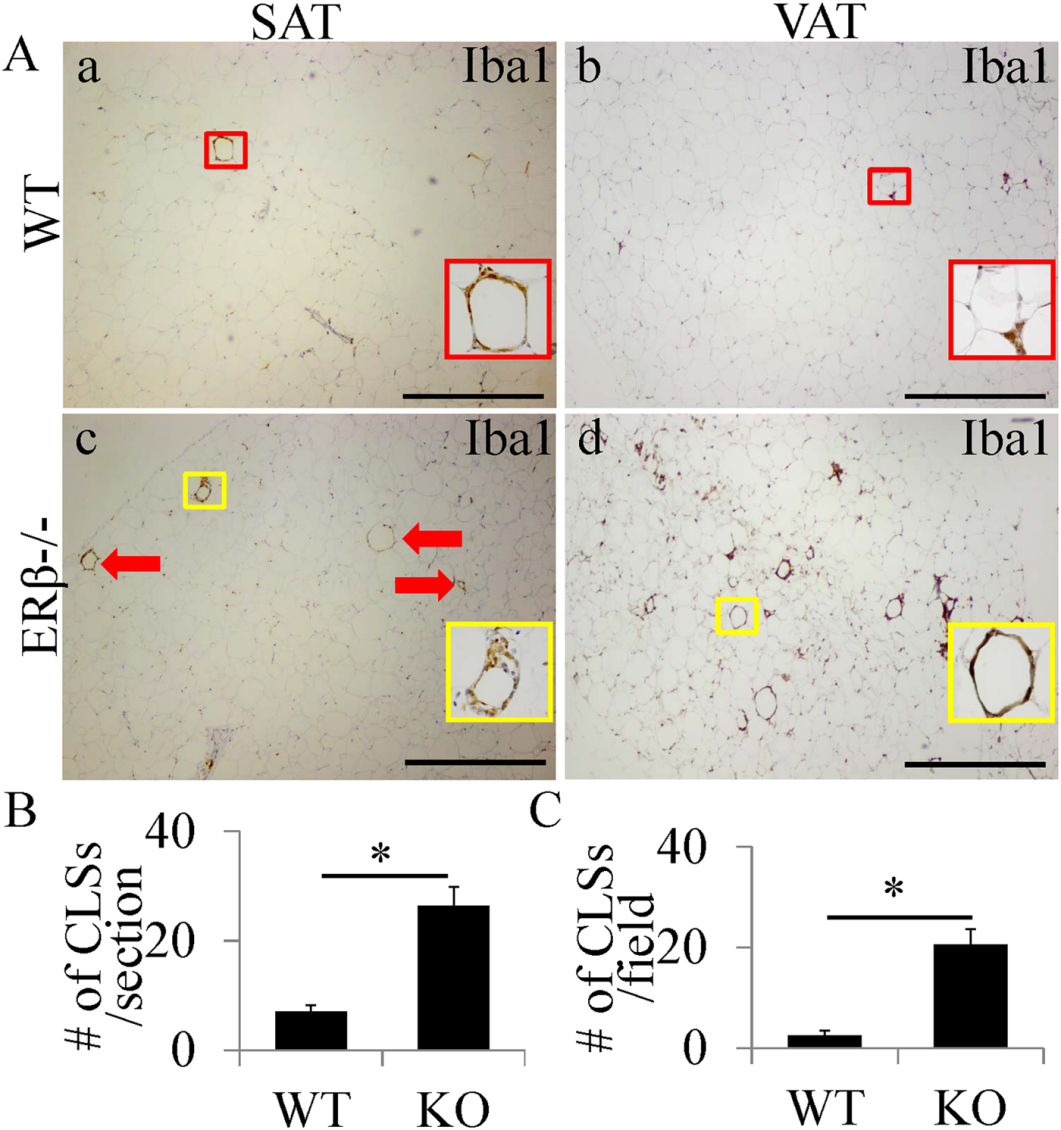
Increase of CLS number in both SAT and VAT of ERβ−/− mice. (**A**) Iba1 staining in SAT and VAT. In WT mice, there were a few CLS (red square) in both SAT and VAT (a,b). In ERβ−/− mice, the number of CLS was much higher (*P < 0.01) than that in SAT or VAT of WT mice (**A**c, d and **B**,**C**). For each picture, a close-up view of a CLS has been included. (Scale bars in A, 500 μm).

To investigate whether there is an increase in the number of CLS in adipose tissue, both SAT (from mammary glands) and VAT (from mesenteric adipose tissue) were analyzed. Because CLS are microscopic foci of dying adipocytes surrounded by macrophages, Iba1 staining was performed to identify CLS. There were a few CLS in both SAT and VAT in 12-month-old WT mice (Fig. 2Aa,b). However, in ERβ−/− mice^29^ there were much more CLS in both SAT and VAT (Fig. 2Ac, Ad and 2B, 2C). To further identify which subtype of macrophages dominated in CLS, we used IHC staining for M1 macrophage makers (IL-1β and iNOS) or M2 macrophage maker (cd206). Macrophages in CLS were strongly stained for M1 macrophage makers (compared to WT mice IL-1β and iNOS staining is much stronger in ERβ−/− mice) with very weak staining for cd206 (Fig. s2). Thus we conclude that CLS are dominated by M1 macrophages. No significant difference in body weight (WT: 33.9 ± 2.99 g vs ERβ−/−: 34.47 ± 2.90 g) was found between WT mice and ERβ−/− mice.

### Macrophages without ERβ express more OPN with higher proliferation

**Figure 3:**
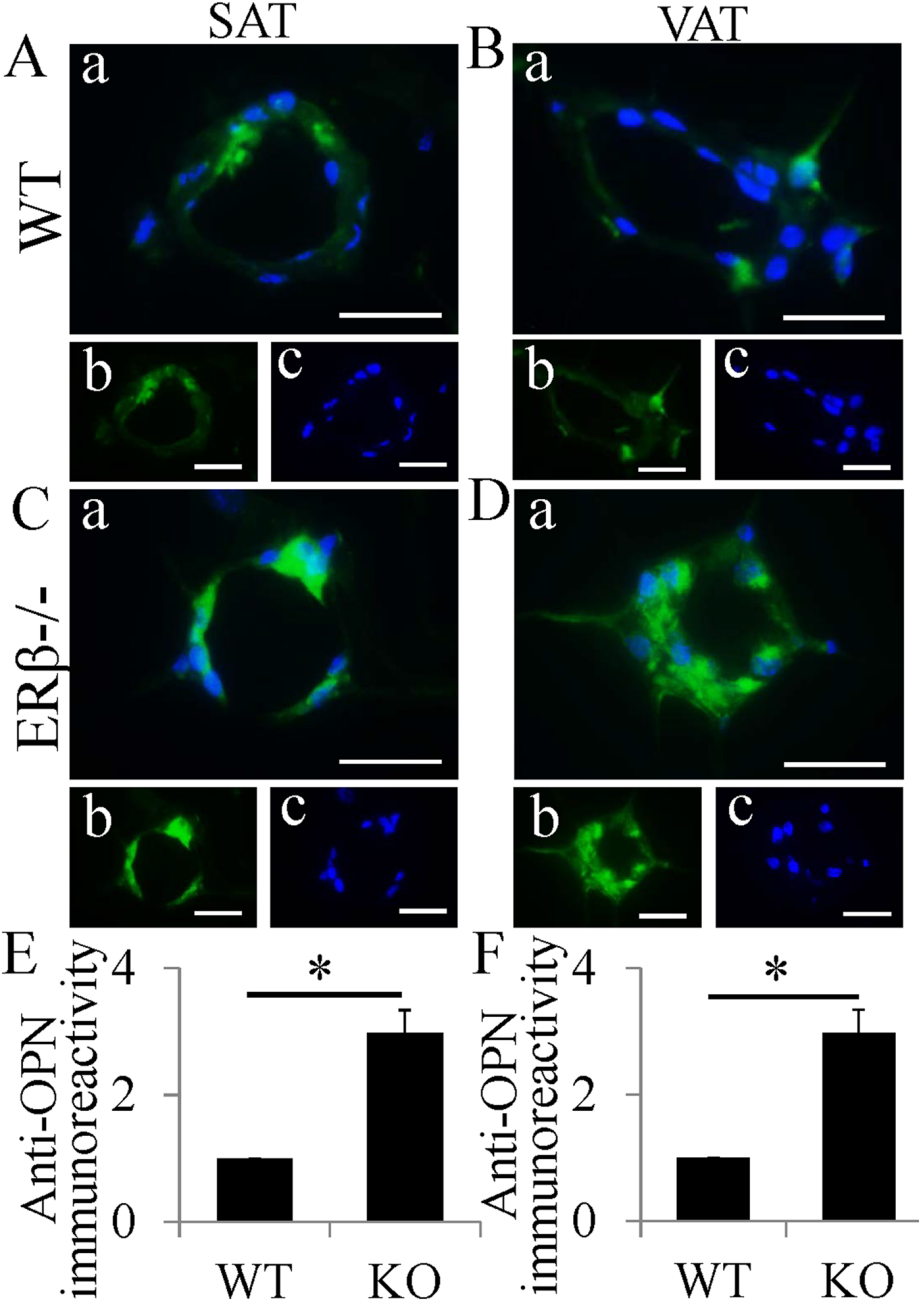
Increased expression of OPN in macrophages in CLS of ERβ−/− mice. (**A–D**) OPN fluorescent staining in SAT and VAT. OPN was expressed in macrophages in CLS of WT mice both in SAT and VAT (A, B). OPN fluorescent intensity was much stronger in macrophages in CLS of ERβ−/− mice (**C,D**). (**E,F**) Expression of OPN was increased about 3 folds in ERβ−/− mice (*P < 0.01). (Scale bars in A–D, 50 μm).

OPN in activated macrophages induced macrophage proliferation in a manner similar to that induced by macrophage colony stimulating factor (M-CSF). Even in fully differentiated macrophages, OPN still induced a proliferative response^19^. We used OPN fluorescent staining to determine whether there was a change in OPN expression in macrophages within CLS. Macrophages in CLS of WT mice did express OPN in cytoplasm (Fig. 3Aa–c,Ba–c), but in ERβ−/− mice, the macrophages had high levels of OPN (Fig. 3Ca–c,Da–c). It was increased about 3-fold in ERβ−/− mice (Fig. 3E,F). We used Ki67 staining to determine whether higher expression of OPN correlated with higher cell proliferation. Only scattered Ki67-positive cells were found in CLS of WT mice (Fig. s3Aa,b). Ki67-positive cells in CLS were clearly higher in the ERβ−/− mouse (Fig. s3Ac,d). Percentages of Ki67-positive cells in the ERβ−/− mouse were significant higher than that in WT mice (Fig. s3B,C). The number of macrophages per CLS was calculated to identify whether there was a difference between WT mice and ERβ−/− mice. In SAT number of macrophages/CLS in WT mice and ERβ−/− mice was 14.17 ± 1.72 and 24.41 ± 2.16 respectively. It was 11.52 ± 1.39 and 17.66 ± 1.85 in VAT. The differences in both SAT and VAT were significant (p < 0.05). These data show that macrophages in CLS of ERβ−/− mice express higher level of OPN and proliferate more than macrophages in CLS of WT mice.

### HIF-1α is unchanged with increased PHD2 in macrophages in CLS of ERβ−/− mice

**Figure 4:**
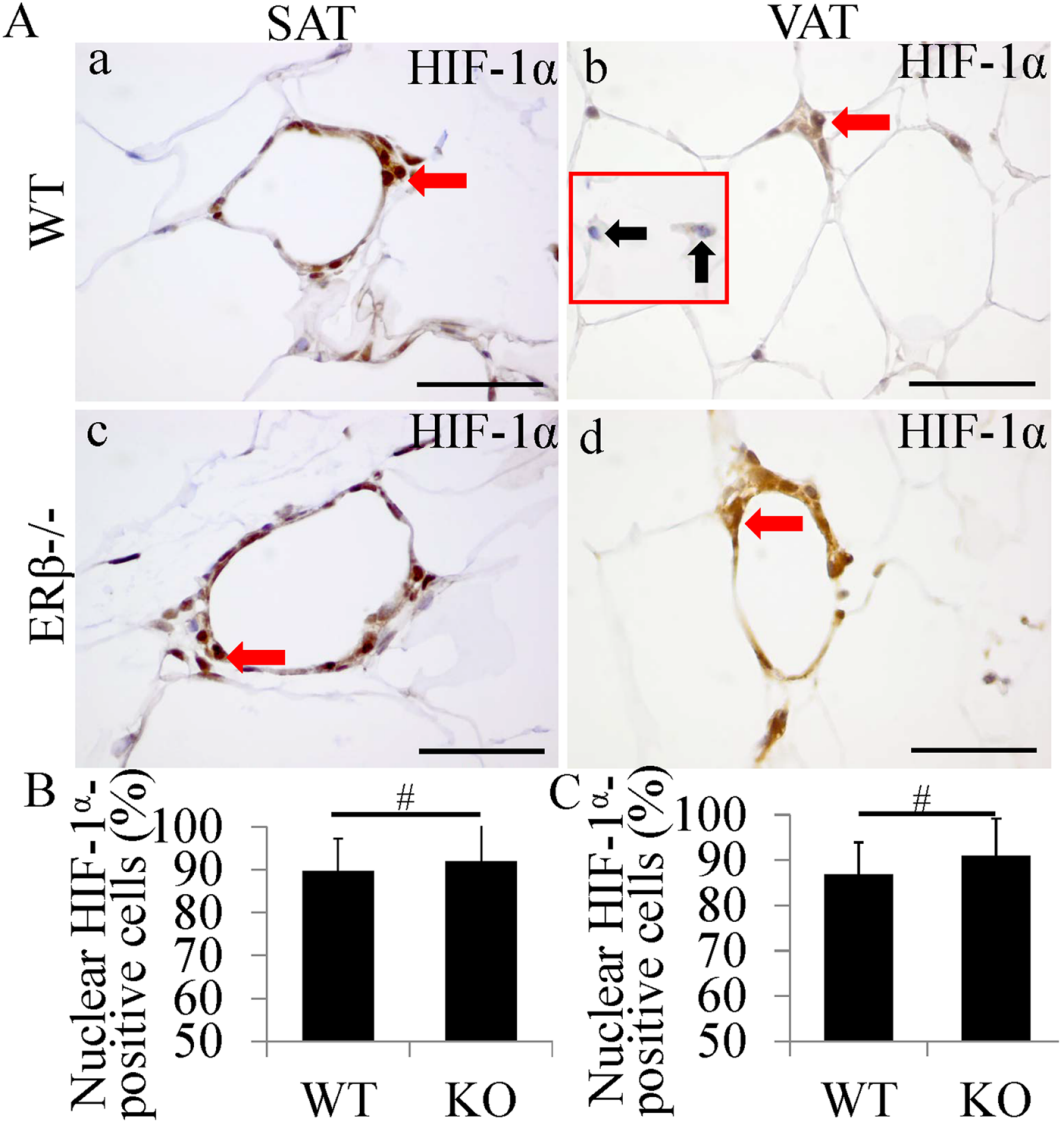
No significant change of HIF-1α expression in macrophages in CLS. (**A**) HIF-1α staining in SAT and VAT. HIF-1α can be detected in both cytoplasm and nucleus (red arrows). HIF-1α stained in both cytoplasm and nucleus of macrophages in WT mice (a,b). In ERβ−/− mice, HIF-1α also stained in both cytoplasm and nucleus of macrophages (c,d). Macrophages outside CLS were not hypoxic in WT mice (black arrows in insert pictures in b). (**B,C**) No significant change in ratio of nuclear HIF-1α-positive cells was found between WT mice and ERβ−/− mice. (Scale bars in Aa–d, 50 μm).

Macrophages in CLS are both hypoxic and inflammatory. HIF-1α in macrophage was involved in the formation of CLS, further enhancing the inflammatory responses^4^. HIF-1α immunohistochemistry confirmed that HIF1a was expressed in both cytoplasm and nuclei of macrophages in WT mice and ERβ−/− mice (Fig. 4Aa–d). This result confirmed that macrophages are hypoxic once CLS are formed. There were no significant differences in percentage of nuclear HIF-1α-positive cells in either SAT (Fig. 4B) or VAT (Fig. 4C). Macrophages outside CLS were not hypoxic in WT mice (black arrows indicate macrophages outside CLS in insert pictures in Fig. 4Ab). It has been reported that ERβ activation increases PHD2 (a HIF inhibitor) and decreased HIF-1α expression. However, we found that PHD2 in macrophages of ERβ−/− mice was 3- to 4-fold higher than that in macrophages of WT mice (Fig. s4). It has been report that these ERβ−/− mice, by 5 months of age, have fewer alveoli and reduced elastic recoil, which leads to systemic hypoxia^30.31^. H&E staining of 12 m-old ERβ−/− mouse lung also showed abnormal lung structure with fewer cells in lung parenchyma and more empty spaces (Fig. s5). This indicates that ERβ−/− mice also have systemic hypoxia. Increased PHD2 in macrophage may be a response to systemic hypoxia.

### An ERβ agonist reduces number of CLS in WD-induced obese mice

**Figure 5:**
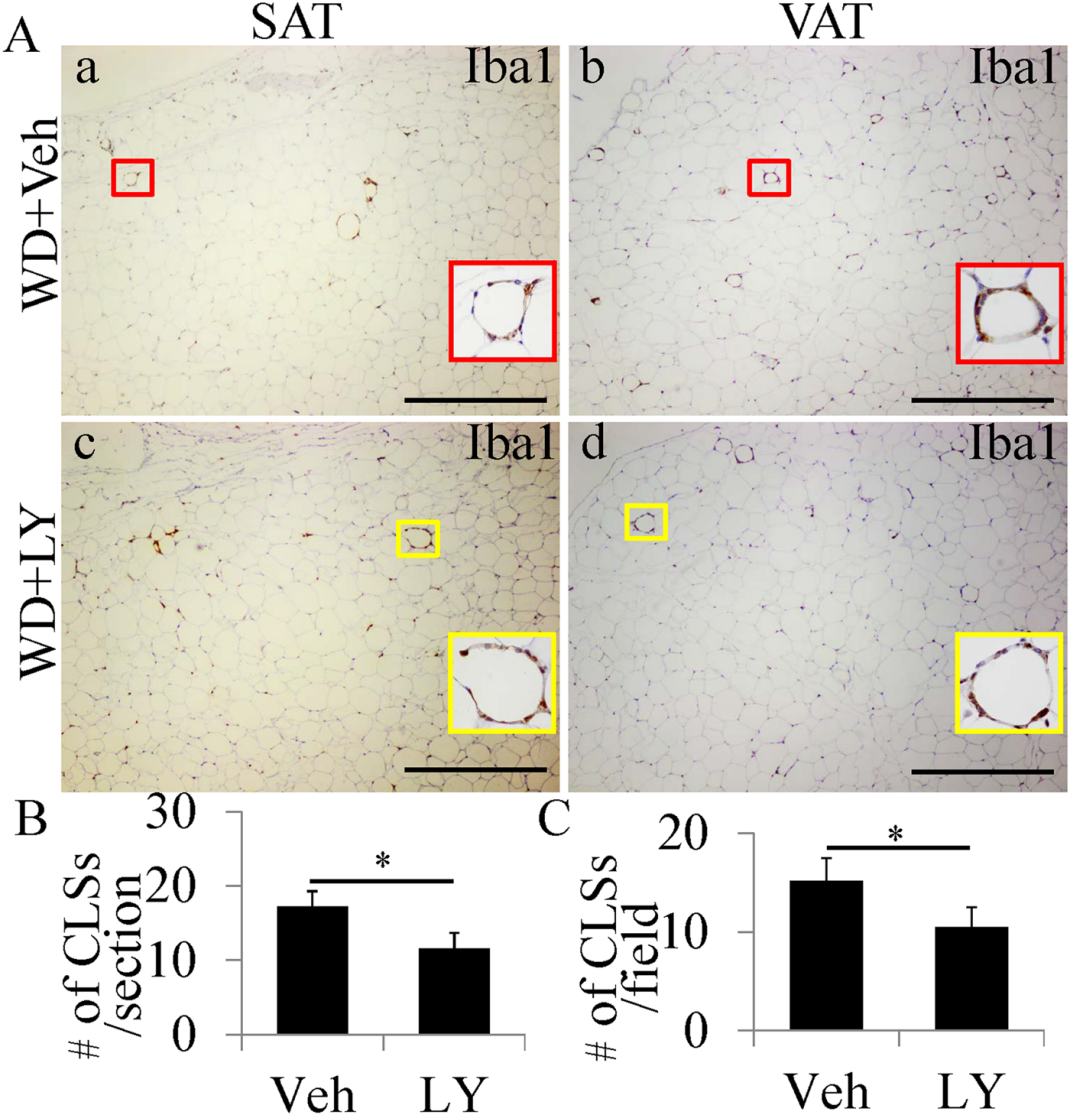
Decrease in CLS number in both SAT and VAT of WD-induced obese mice by LY3201 treatment. (**A**) Iba1 staining in SAT and VAT. In vehicle-treated mice, there were many CLS in both SAT and VAT (a,b). There were much less CLS in both SAT and VAT of LY3201-treated mice (c,d). (**B**,**C**) LY3201 treatment significantly decreased CLS number in both SAT and VAT (*P < 0.01). For each picture, a close-up view of a CLS has been included. (Scale bars in A, 500 μm).

ERβ has anti-inflammatory actions and an ERβ agonist has been used to repress activation of microglia in the CNS^27,28^. LY3201 administration to mice resulted in a marked reduction of CLS number in both SAT and VAT of WD-induced obese mice (Fig. 5Aa–d,B,C). In addition, expression of the proinflammatory cytokine IL-1β, and TNFα in macrophages was also downregulated in CLS by LY3201 treatment (Fig. s6). No significant difference in body weight (Veh: 41.1 ± 2.04 g vs LY: 39.8 g ± 1.92 g) was found between vehicle-treated mice and LY3201-treated mice.

### An ERβ agonist inhibits macrophage proliferation with down-regulating osteopontin

**Figure 6:**
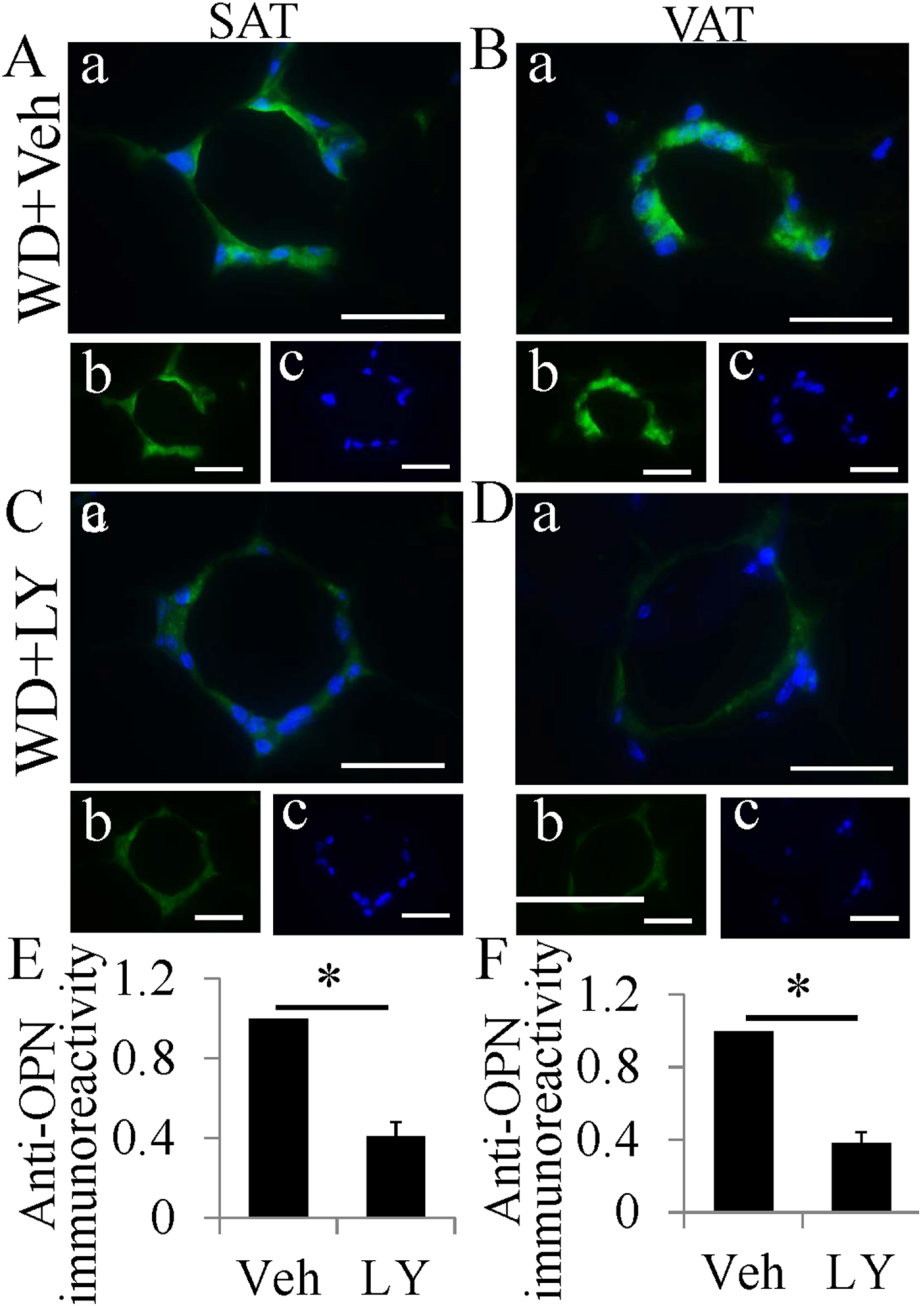
Downregulation of OPN expression in macrophages in CLS by LY3201 treatment. (**A–D**) OPN fluorescent staining in SAT and VAT. Macrophages in CLS of vehicle-treated mice expressed high level of OPN both in SAT and VAT(**A,B**). OPN staining was much weaker in macrophages in CLS of mice treated by LY3201(**C,D**). (**E,F**) Expression of OPN was downregulated about 60% by LY3201 (*P < 0.01). (Scale bars in A-D, 50 μm).

In ERβ−/− mice, macrophages in CLS expressed much higher level of OPN than that in WT mice. LY3201 treatment significantly downregulated OPN expression in macrophages in CLS (Fig. 6A–F). Overall LY3201 administration resulted in reduction in OPN expression and loss of ERβ resulted in increased OPN expression. Ki67 immunohistochemistry was performed to investigate whether LY3201 repressed cell proliferation. We demonstrated that after seven days of LY3201 treatment, there was a significant decrease in ratio of Ki67-positive cells in CLS (Fig. s7). The number of macrophages per CLS was calculated to identify whether LY3201 treatment decreased it. In SAT number of macrophages/CLS in vehicle-treated mice and LY3201-treated mice was 15.31 ± 1.96 and 8.73 ± 1.53 respectively. In VAT it was 16.29 ± 1.62 and 9.48 ± 1.75. LY3201 treatment significantly decreased the number of macrophages/CLS (p < 0.05).

### An ERβ agonist decreases HIF-1α expression and nuclear translocation with up-regulating PHD2 in macrophages

**Figure 7:**
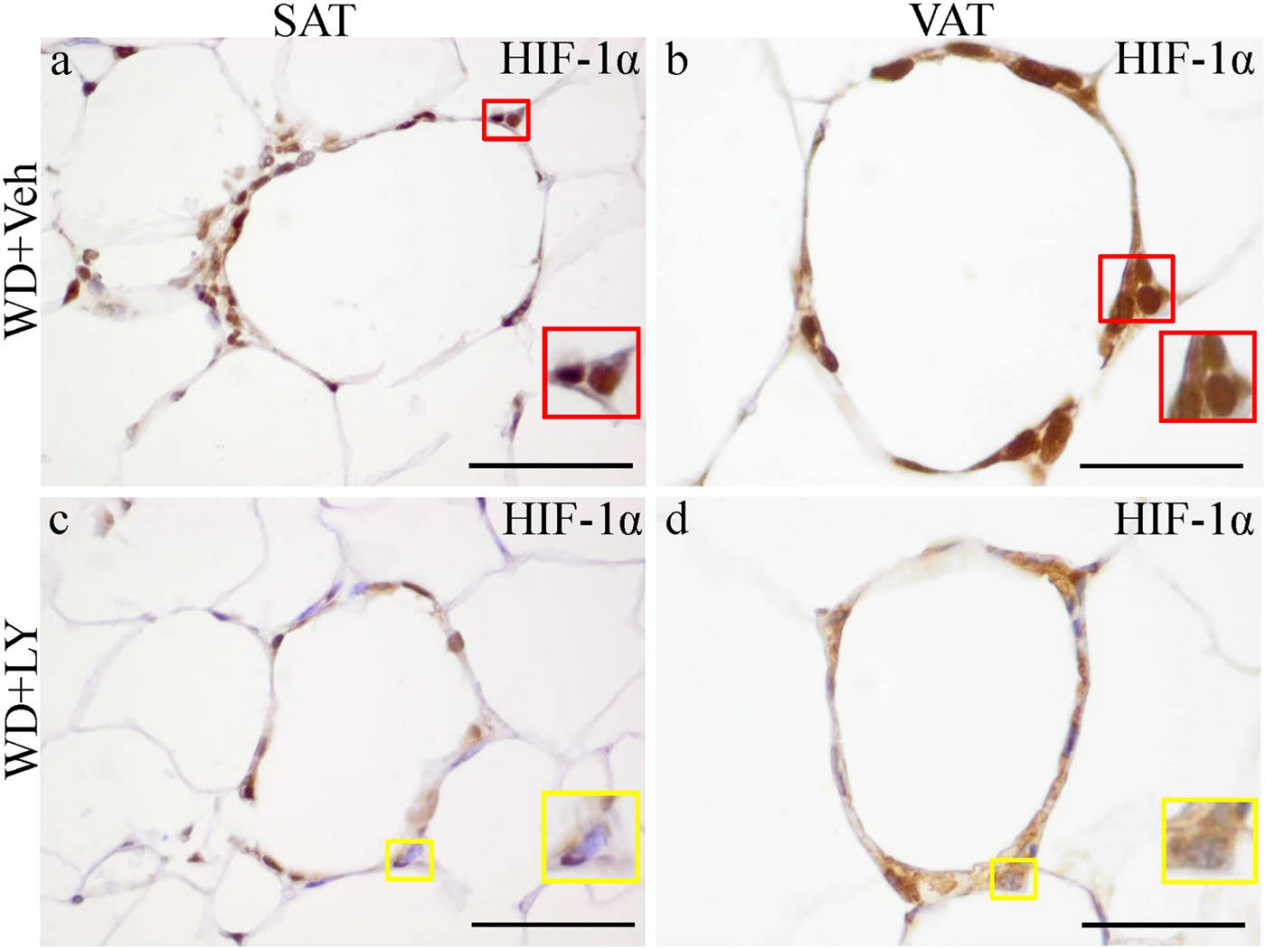
Decrease in HIF-1α expression and nuclear translocation in macrophages upon treatment with LY3201. There was strong HIF-1α expression in both cytoplasm and nuclei in macrophages of the vehicle-treated mice (red arrows) (**a,b**). LY3201 downregulated HIF-1α cytoplasm expression and obviously decreased nuclear HIF-1α (**c,d**). For each picture, a close-up view has been included. (Scale bars in a–d, 50 μm).

In vehicle-treated mice, macrophages highly expressed HIF-1α in both the cytoplasm and nucleus (active HIF-1α) (Fig. 7a,b). LY3201 treatment downregulated HIF-1α cytoplasm expression and caused a clear decreased nuclear HIF-1α (Fig. 7c,d). In addition, we demonstrated that PHD2 expression was significantly upregulated in macrophages by LY3201 treatment (Fig. s8). Overall LY3201 treatment inhibits HIF-1α expression and up-regulates PHD2 in macrophages within CLS.

### Activation of ERβ does not reduce number of CLS or inhibit macrophage proliferation in ERβ−/− mice

**Figure 8:**
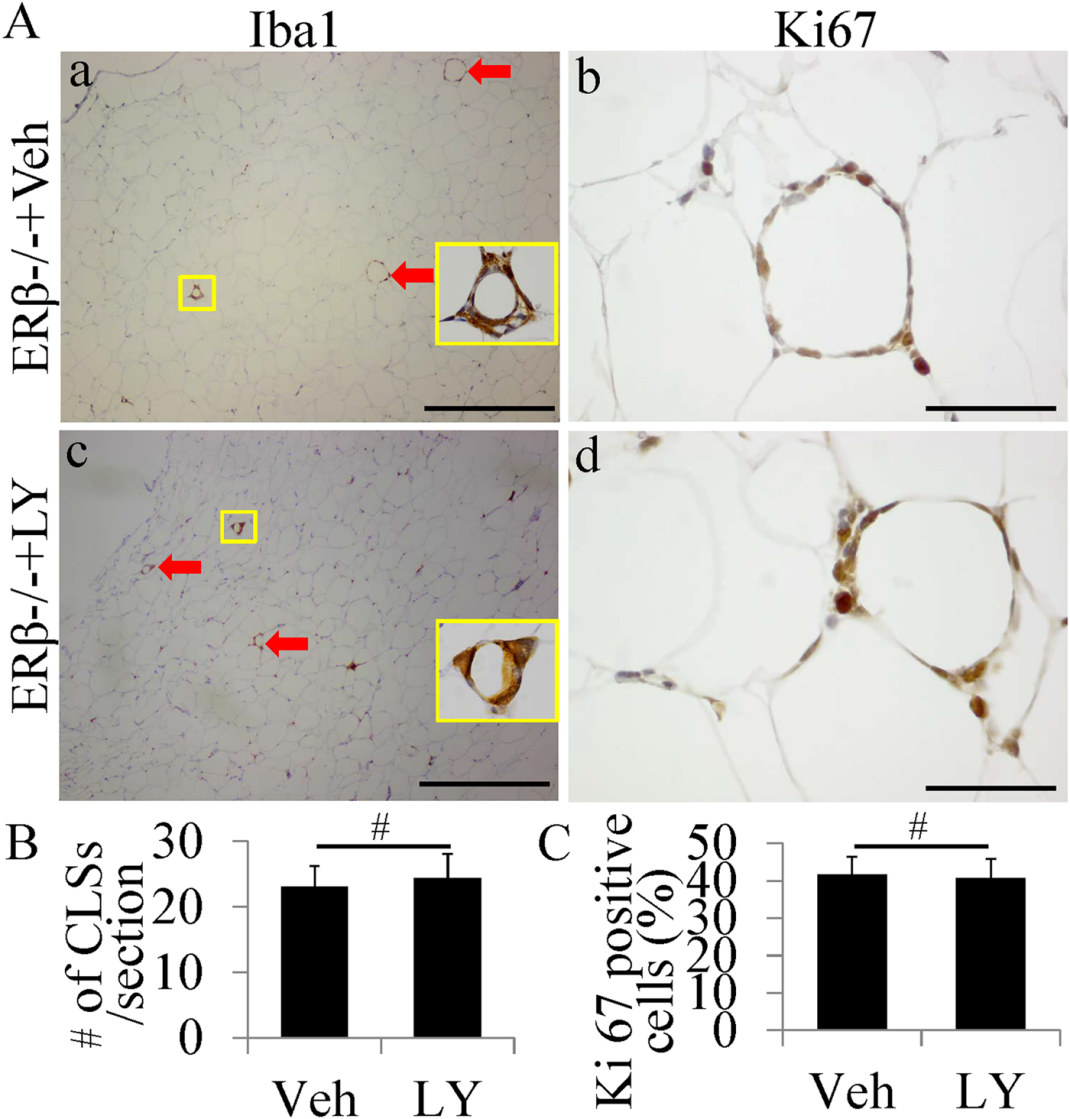
No change of CLS Number or Macrophage Proliferation in ERβ−/− mice treated by LY3201. (**A**) Iba1 and Ki67 staining in SAT. In either vehicle-treated or LY3201-treated ERβ−/− mice, there were many CLS in SAT (a,c). Ki67-positive cells were found in CLS in SAT of either vehicle-treated or LY3201-treated ERβ−/− mice (b,d). (**B,C**) No significant difference was found in CLS number or in ratio of Ki67-positive cells in CLS between vehicle-treated and LY3201-treated ERβ−/− mice. (Scale bars in a,c, 500 μm; b,d, 50 μm).

To further confirm that the effect of LY3201 on macrophage in CLS was mediated by ERβ, we treated 6-month old ERβ−/− mice with vehicle or LY3201. In the SAT of ERβ−/− mice LY3201 treatment failed to reduce the number of CLS (Fig. 8Aa,c,B) or to inhibit macrophage proliferation (Fig. 8Ab,d,C). These results suggested that LY3201 is an effective ERβ-selective agonist, and activation of ERβ is responsible for LY3201-caused decreasing in number of CLS and inhibiting of macrophage proliferation in CLS in adipose tissues.

## Discussion

In the present study, we demonstrated that mice without ERβ have a significant increase of CLS number in both SAT and VAT. Macrophages in CLS in ERβ−/− mice express higher OPN and proliferate more. LY3201 (an ERβ selective agonist) treatment reduces the number of CLS in adipose tissue with downregulating OPN and HIF-1α in macrophages of mice made obese by Western-diet. Furthermore, we demonstrate LY3201 inhibits macrophage proliferation and upregulates PHD2, the enzyme which prevents HIF-1α activation.

CLS, accumulation of macrophages that surround dead or dying adipocytes, are markers of chronic inflammation in adipose tissues. With increased body mass index (BMI) CLS number is increased in postmenopausal women^32^. CLS were reported associating with obesity and a proinflammatory, pro-carcinogenic process^33^. Presence of CLS negatively correlates with outcomes in female with ERα-positive breast cancers^8^. CLS-related carcinogenic processes can be reversed by weight-loss or chemopreventive agents in mice^34,35^. Macrophages within CLS in adipose tissue are also known to play important roles in the pathophysiology of IR and to be related to systemic IR^6,36^. Thus reducing CLS may be used to alleviate CLS-related breast cancer and insulin resistance.

Macrophages are the main components of CLS. It has been reported that macrophages in mice express ERα^37,38^. In the present study, we found with immunohistochemistry and immunofluorescence that ERβ is expressed in mouse macrophages even though it is not detectable in monocytes in the blood. This result confirmed that ERβ selective agonist is able to target macrophage directly. It has been reported that hypoxia increased ERβ, but not ERα expression in both human and rat pulmonary artery endothelial cells (PAECs)^39^. ERβ mRNA is time-dependently increased when PAECs were exposed to hypoxia. ERβ expression in PAEC is increased by normoxic HIF-1α stabilization, while ERβ expression in hypoxic PAECs is decreased by HIF-1α knockdown^39^. The present study demonstrated either macrophages in CLS in WT mice or in ERβ−/− mice expressed HIF-1α in nuclei (active HIF-1α). It also showed that macrophages are hypoxic in CLS. More studies are needed to investigate whether ERβ in macrophages within CLS is also induced by hypoxia through HIF signaling.

Two polarized phenotypes, pro-inflammatory macrophages (classically activated M1 macrophages) and constructive macrophages (alternatively activated M2 macrophages), of activated macrophages have been identified^40^. M1 macrophages are involving in inflammatory diseases and host’s defense against infection^41^. M2 macrophages are relating to tissue remodeling and inflammation relief^42^. In the present study, we found that in CLS the dominant macrophages were M1 macrophages with high expression of IL-1β and iNOS. LY3201 administration not only decreased the number of CLS but also significantly inhibited inflammatory factors in macrophages within CLS.

OPN expressed by activated macrophages has an important role in cell-mediated immunity^16^. OPN played a key role in the development of insulin resistance through promoting macrophage accumulation and enhancing inflammation in adipose tissue^43^. Moreover, even in fully differentiated macrophages OPN still induces macrophage proliferation^19^. In this study, our results show that macrophages in CLS of ERβ−/− mice expressed much higher level of OPN with higher cell proliferation. Thus high expression of OPN in the ERβ−/− mouse may promote macrophage proliferation. Furthermore, LY3201 treatment significantly repressed macrophage proliferation with downregulated OPN expression. Overall LY3201 treatment resulted in reduction in OPN expression and lacking ERβ resulted in upregulated OPN expression. This result gives a possible explanation for the anti-proliferative effect of ERβ in macrophages within CLS.

CLS formed by HIF-1α-activated macrophages are the major sites of hypoxia in later stages of obesity^24^. M1 polarization of macrophages is further induced by HIF-1α activation^25^. Thus, HIF-1α in macrophages may be a potential therapeutic target for CLS-related diseases. In hypoxic PAECs, knockdown ERβ leads to a decrease expression of PHD2 (HIF-1α inhibitor), while activation of ERβ causes an increase of PHD2 and decreases HIF-1α. These data suggest that PHD2/HIF-1α axis is regulated by ERβ during hypoxia^39^. Either macrophage in WT mice or in ERβ−/− mice expressed HIF-1α in both cytoplasm and nuclei indicating that macrophages are hypoxic once CLS are formed. However, the interesting finding in this study is that PHD2 in macrophages was upregulated in ERβ−/− mice. To explain this unexpected finding, H&E staining of the ERβ−/− mouse lung was performed. Abnormal lung structure with less cells and more empty spaces was found. This indicates that ERβ−/− mice have systemic hypoxia. Increased PHD2 in macrophage may be a response to systemic hypoxia. However, in diet-induced obese mice, LY3201 treatment significantly decreased HIF-1α expression in both cytoplasm and nuclei in macrophages. We further demonstrated that HIF-1α expression inhibited by LY3201 was mediated though up-regulated PHD2 in macrophages within CLS.

Six-month old ERβ−/− mice were treated with vehicle or LY3201 to confirm that the effect of LY3201 on macrophage in CLS is mediated by ERβ. No effect was found on the number of CLS or on proliferation of macrophage. It proved that activation of ERβ is responsible for LY3201-induced decreasing in number of CLS and inhibiting of macrophage proliferation in CLS. In our previous study^44^, to rule out LY3201 acting through ERα, ERα−/− female mice at 1-year-old age were treated by LY3201 for 3 days. Similar to WT mice, LY3201 treatment still increased browning of SAT without correcting the abnormalities in the ovary and uterus of ERα−/− mice. Based on the previous finding, the effect of LY3201 in this study is not mediated by ERα even there is ERα in macrophages.

Overall our data prove that ERβ plays a key role for in CLS formation by regulating OPN and HIF-1α in macrophages within CLS and suggest that stimulating ERβ in macrophage should be useful in treating CLS-related disorders.

## Materials and Methods

### Materials and animals

LY3201 (3aS, 4 R, 9bR)-2, 2-difluoro-4-(4-hydroxyphenyl)-3, 3a, 4, 9b-tetrahydro-1H-cyclopenta[c] chromen-8-ol (CAS 787621–78-7), an ERβ agonist, was from Eli Lilly. The animal studies were approved by the local Animal Experimentation Ethics Committee for animal experimentation (University of Houston animal protocol 09-036). All experimental protocols were adhered to the National Institutes of Health Guidelines for the Care and Use of Laboratory Animals. Effort was made to minimize the number of mice and their suffering. In this study, 12-month old ERβ−/− (B6.129P2-Esr2tm1Unc/J, The Jackson Laboratory) and their littermate female mice (five mice for each group) and 5-month old C57BL/6 male mice were used for experiments. Ten C57BL/6 male mice were induced to be obese by Western diet (Research Diets Inc. NJ, USA. Cat#: D12079B. Its caloric composition is 17 kcal% protein, 43 kcal% carbohydrate, and 40 kcal% fat. Every gram of its ingredient contains 4.67 kcal calorie) and then randomly separated into 2 groups: (1) Mice treated with Vehicle (n = 5); (2) Mice treated with LY3201 (n = 5). To confirm that the effect of LY3201 on CLS was mediated by ERβ 6-month old ERβ−/− (B6.129P2-Esr2tm1Unc/J, The Jackson Laboratory) female mice were treated by either vehicle (n = 4) or LY3201 (n = 4). LY3201 was used as pellets (0.04 mg/d) made by Innovative Research of America (2 North Tamiami Trail, Suite 404. Sarasota, Florida 34236)^44,45^ and implanted on the lateral side of the neck between the ear and the shoulder. The mice were treated by either vehicle pellets or LY3201 pellets for 7 days. All mice were anesthetized by CO_2_ and transcardially perfused with 1XPBS followed by 4% paraformaldehyde (in 0.1 M PBS, pH7.4). SAT and VAT were dissected and postfixed in 4% paraformaldehyde overnight at 4 °C. Tissues were processed for paraffin sections at thickness of 5 μm after fixation.

### Immunohistochemistry

Paraffin-embedded sections were processed for antigen retrieval with 10 mM citrate buffer (pH 6.0) in a Lab Vision PT module (Thermo Scientific) After dewaxed in xylene, rehydrated. Buffer composed of 50% (vol/vol) methanol and 3% (vol/vol) H_2_O_2_ was used to quench endogenous peroxidase and then 3% (wt/vol) BSA with 0.1% Nonidet P-40 in PBS was used to block unspecific binding. Sections were then incubated with anti-ERβ (1:100; in house ERβ antibody mapping the C-terminus part^46–48^), anti-Iba1 (1:5000; Abcam), anti-IL-1β (1:100; Abcam), anti-IL-6 (1:50; Abcam), anti-TNFα (1:100; Abcam), anti-iNOS (1:100; Millipore), anti- Arginase1(1:100; Abcam), anti-Cd206 (1:100; Abcam), anti-Ki67 (1:2000; Abcam), anti-HIF-1α (1:100; Novus Biologicals), and anti-PHD2 (1:100; Novus Biologicals) at 4 °C for overnight. In negative controls primary antibodies were replaced by 3% BSA. Sections were incubated with HRP polymer kit (Biocare Medical; GHP516) for 30 min after washing, followed by 3, 3-diaminobenzidine tetrahydrochloride as the chromogen. Immunofluorescence was performed as described before^31^. Sections were incubated overnight with anti-OPN(1:100; Abcam), anti-Cd11b (1:100; Abcam) and anti-ERβ (1:100; in house) or anti-Iba1 (1:5000; Abcam) and anti-ERβ (1:100; made in our laboratory) at 4 °C after blocking nonspecific binding in 3% BSA. Primary antibodies were detected with donkey anti rabbit FITC (1:400; Jackson Immuno Research), donkey anti chicken FITC (1:400; Jackson Immuno Research) and donkey anti mouse Cy3 (1:400; Jackson Immuno Research), donkey anti chicken Cy3 (1:400; Jackson Immuno Research) and donkey anti rabbit FITC (1:400; Jackson Immuno Research). To label nuclei sections were counterstained with Vectashield mounting medium containing 4′, 6′-diamidino-2-phenylindole (DAPI) (Vector). Every fifth slide from 25 consecutive slices i.e., five slices from each mouse were stained. The ImageJ software was used to quantitate of levels of immunoreactivity. Before statistic analyze, wild type mice or vehicle-treated mice were set as standard.

### Data analysis

Data are expressed as mean ± SD; GraphPad Prism 5.0 (GraphPad Software, Inc., La Jolla, CA, USA) was used for statistical analysis. Statistical comparisons were made by using a one-way ANOVA followed by the Newman-Keuls post hoc test or using Kruskal-Wallis test followed by Dunns post test or using Paired t-Test. P < 0.05 was considered to indicate statistical significance. The investigators who analyzed the imaging data were blinded to sample condition.

## Supplementary information

supplementary info
