# Supplementary material for: Targeting ERβ in Macrophage Reduces Crown-like Structures in Adipose Tissue by Inhibiting Osteopontin and HIF-1α
Source: Sci Rep. 2019 Oct 31;9:15762. doi: 10.1038/s41598-019-52265-8 (PMC6823357; doi:10.1038/s41598-019-52265-8)
Supplement: Supplementary file 1 — supplementary info [file 41598_2019_52265_MOESM1_ESM.pdf]

Targeting ERβ in Macrophage Reduces Crown-like Structures in Adipose Tissue by Inhibiting Osteopontin and HIF-1α

Li Wang[1], Rui-peng Zhao[2], Xiao-yu Song[1], Wan-fu Wu[1]

Supplementary Information

Figure Legends

Figure s1: Verification of ERβ antibody with HeLa cells transfected with vehicle, ERβ1, ERβ2 or ERα and SAT of WT or ERβ-/- mice. (**A**) ERβ staining in HeLa cells. Only HeLa cells transfected with ERβ1 were stained by ERβ antibody (b). HeLa cells transfected with vehicle, ERβ2 or ERα did not pick up the staining (a,c,d). (**B**) ERβ staining in SAT of WT or ERβ-/- mice. ERβ stained macrophages in CLS in SAT of WT mice (a). There was no detectable ERβ staining in macrophages in CLS of ERβ-/- mice (b). Red arrows show macrophage in CLS. (Scale bars in A,B, 50 μm.)

Figure s2: Expression of makers for M1 and M2 macrophages in CLS. (**A, B**) IL-1β or iNOS (M1 macrophage makers) staining in CLS of WT and ERβ-/- mice. Both IL-1β (Aa and b) and iNOS (Ba and b) expressed in CLS. Expression of IL-1β and iNOS in CLS of ERβ-/- mice is stronger that in WT mice (Ab and Bb). (C) Cd206 (M2 macrophage maker) staining in CLS. Very few Cd206 positive could be found in CLS of WT mice or ERβ-/- mice. Insert pictures showed macrophages outside CLS were CD206 positive (Ca and b). (Scale bars in A-C, 50 μm.)

Figure s3: More proliferating macrophages in CLS of ERβ-/- mice. (**A**) Ki67 staining in SAT and VAT. A few Ki67-positive cells were identified in CLS of WT mice both in SAT and VAT (a,b). In ERβ-/- mice, much more Ki67-positive cells than that in CLS of WT mice (c,d). (**B, C**) In ERβ-/- mice, ratio of Ki67-positive cells was much higher (*P<0.01) than that of WT mice. (Scale bars in A, 50 μm.)

Figure s4: Increased PHD2 expression in macrophages in CLS of ERβ-/- mice. (**A**) PHD2 staining in SAT and VAT. PHD2 was expressed in macrophages in CLS of WT mice (a,b). PHD2 staining was much stronger in macrophages in CLS of ERβ-/- mice

(c,d). (**B, C**)In ERβ-/- mice, expression of PHD2 was increased about 4 folds and 3 folds in SAT and VAT respectively (* P<0.01). (Scale bars in A, 50 μm.)

Figure s5: Abnormal lung structure of ERβ-/- mice. Hematoxylin/eosin staining of WT mouse showed normal alveolar structure in lung (a,b). In ERβ-/- mice, areas of collapsed alveoli were identified (red stars) (c,d). (Scale bars in a and c, 500 μm; b and d, 50 μm.)

Figure s6: Inhibition of IL-1β and TNFα in macrophages by LY3201. (**A, C**) IL-1β and TNFα staining in SAT and VAT. Both IL-1β and TNFα strongly expressed in vehicle-treated mice(**A**a and b**, C**a and b). There was a sharp decrease of IL-1β and TNFα expression in LY3201-treated mice(**A**c and d**, C**c and d). (**B, D**) LY3201 treatment downregulated IL-1β and TNFα expression about 65% and 60% respectively (* P<0.01). (Scale bars in A, B, 50 μm.)

Figure s7: Less proliferating macrophages in CLS of mice treated by LY3201. (**A**) Ki67 staining in SAT and VAT. Some Ki67-positive cells were found in CLS in SAT and VAT of mice treated by vehicle (a,b). In LY3201 treated mice, much less Ki67-positive cells than that in vehicle-treated mice(c,d). (**B, C**) In LY3201 treated mice, ratio of Ki67-positive cells was significantly higher (*P<0.01) than that of WT mice. (Scale bars in A, 50 μm.)

Figure s8: Upregulation of PHD2 expression in macrophages upon treatment with LY3201. (**A**) PHD2 staining in SAT and VAT. In vehicle-treated mice, PHD2 was expressed in macrophages in CLS(a,b). PHD2 expression was upregulated in macrophages by LY3201 treatment(c,d). (**B,C**) LY3201 treatment upregulated PHD2 expression about 4 folds and 3 folds in SAT and VAT respectively (* P<0.01). (Scale bars in A, 50 μm.)

Figure s1

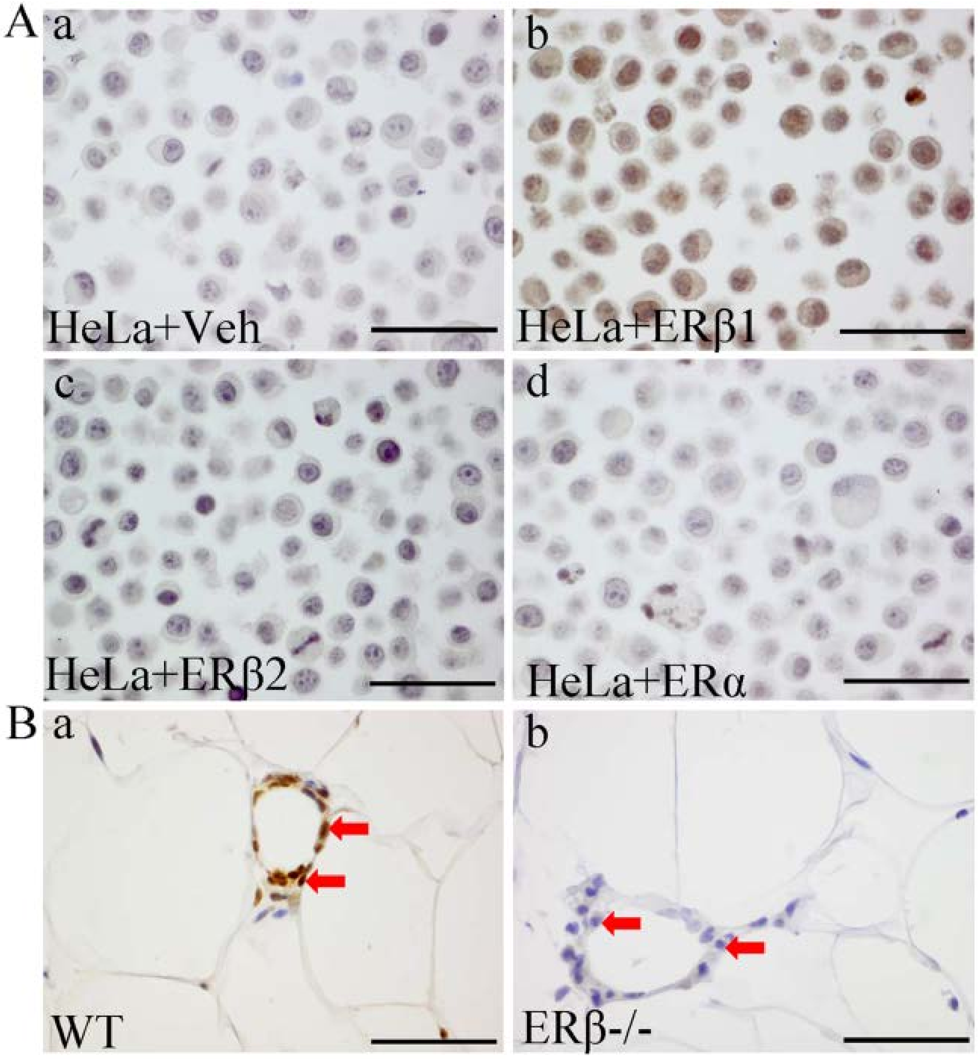

Figure s2

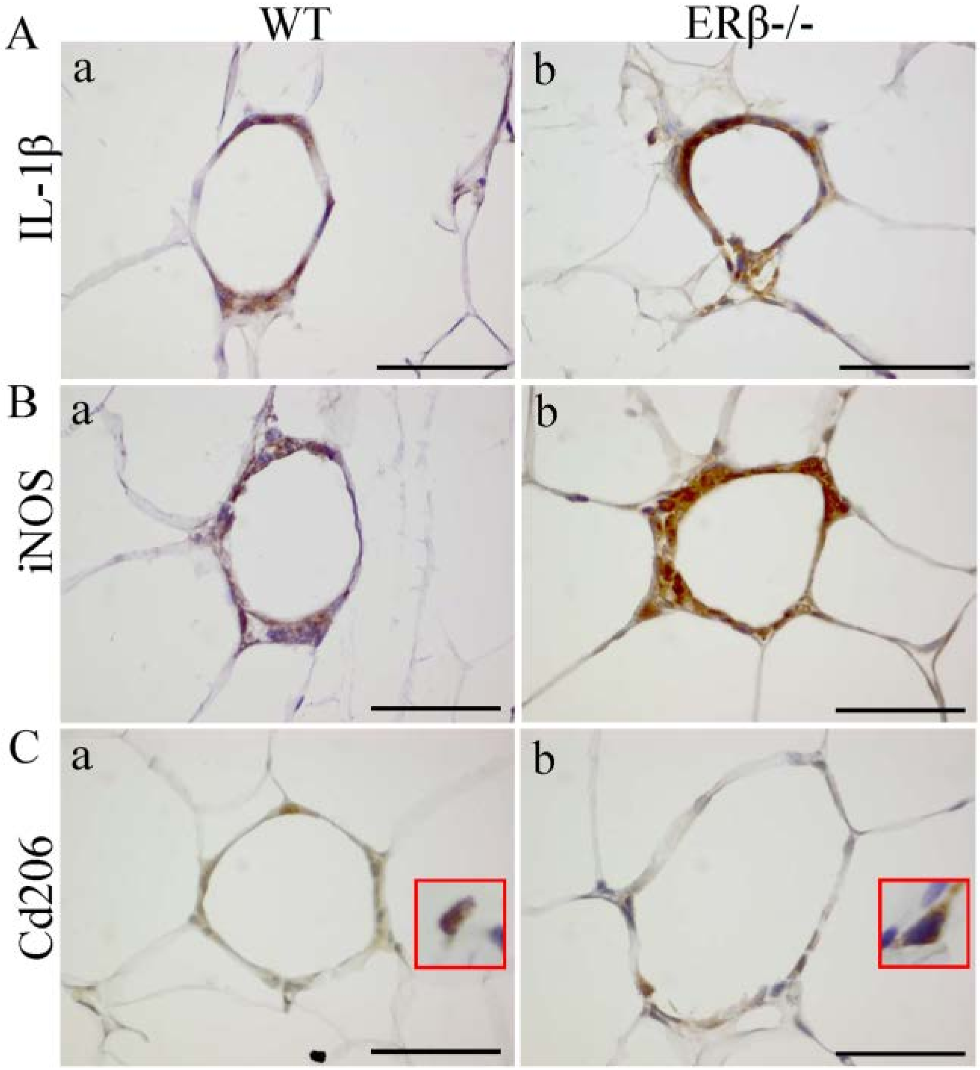

Figure s3

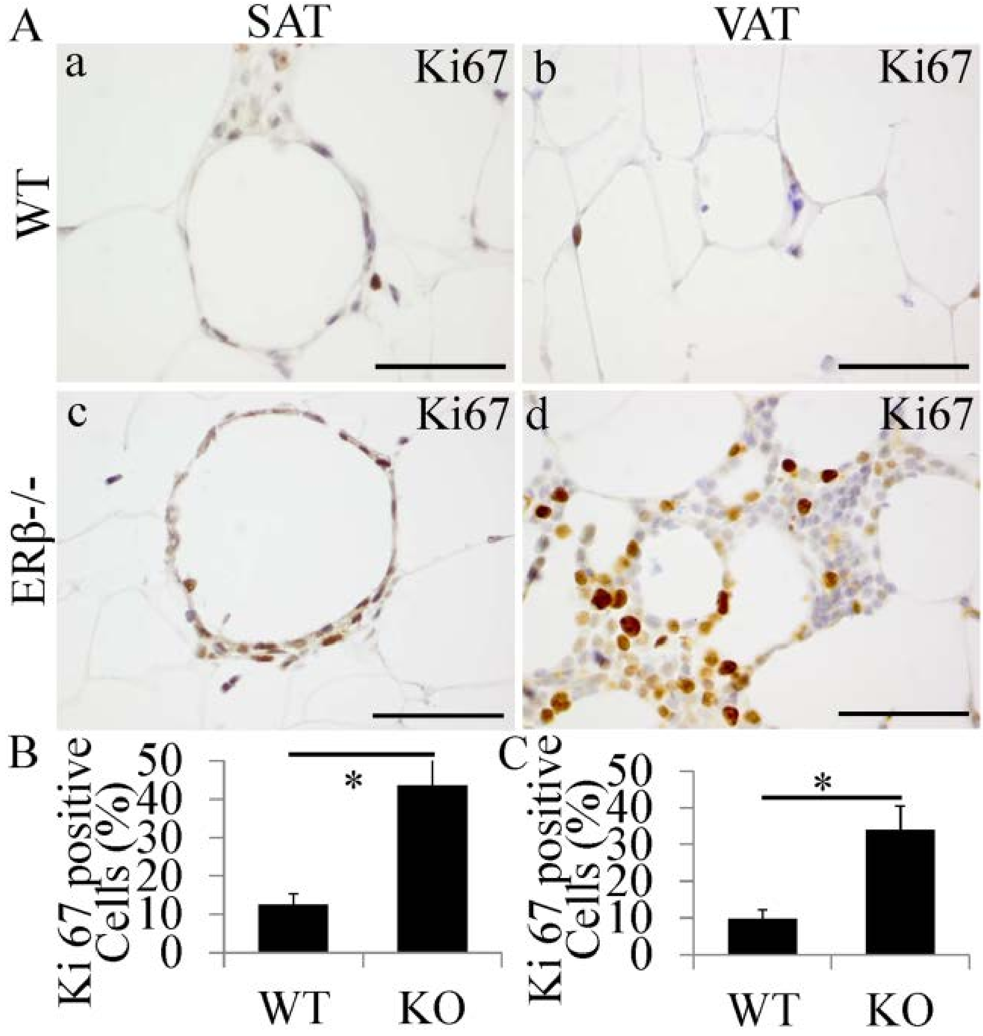

A
SAT
VAT
a
Ki67
b
Ki67
WT
c
Ki67
d
Ki67
ERβ-/-
B
Ki 67 positive Cells (%)
50
40
30
20
10
0
*
WT
KO
C
Ki 67 positive Cells (%)
50
40
30
20
10
0
*
WT
KO

Figure s4

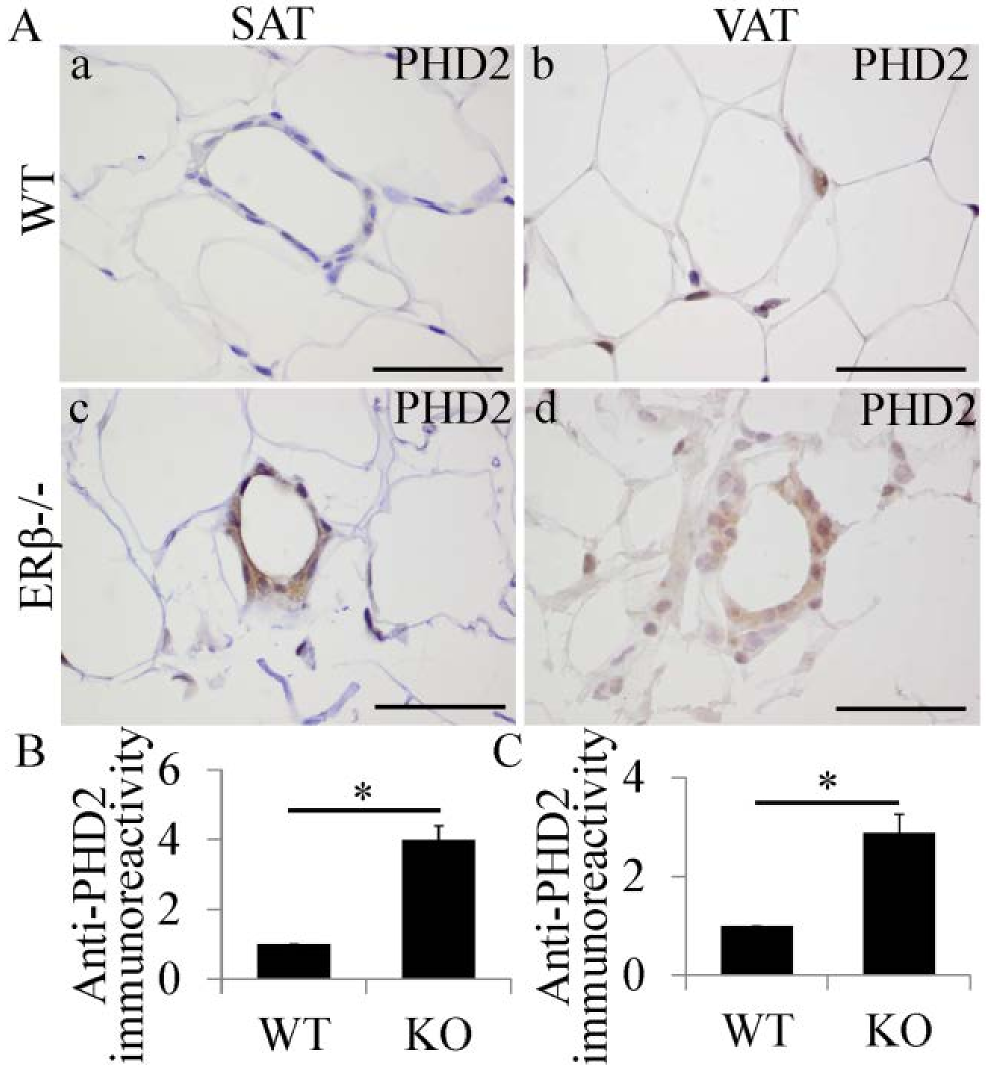
A
SAT
VAT
a
PHD2
b
PHD2
WT
c
PHD2
d
PHD2
ERβ-/-
B
Anti-PHD2 immunoreactivity
6
4
2
0
*
WT
KO
C
Anti-PHD2 immunoreactivity
4
2
0
*
WT
KO

Figure s5

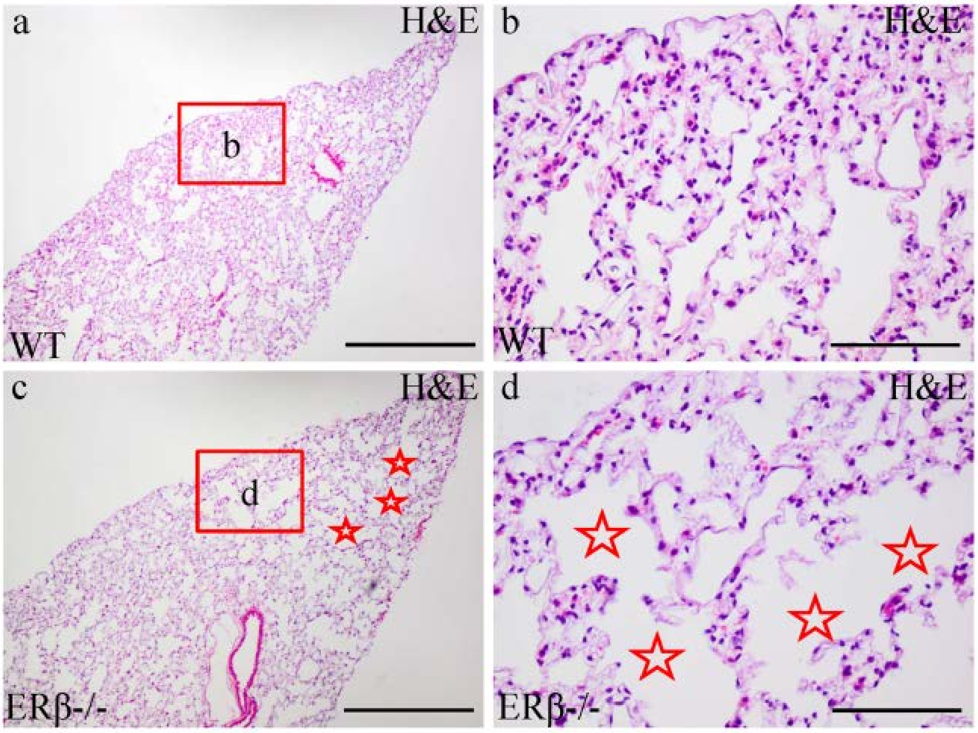

Figure s6

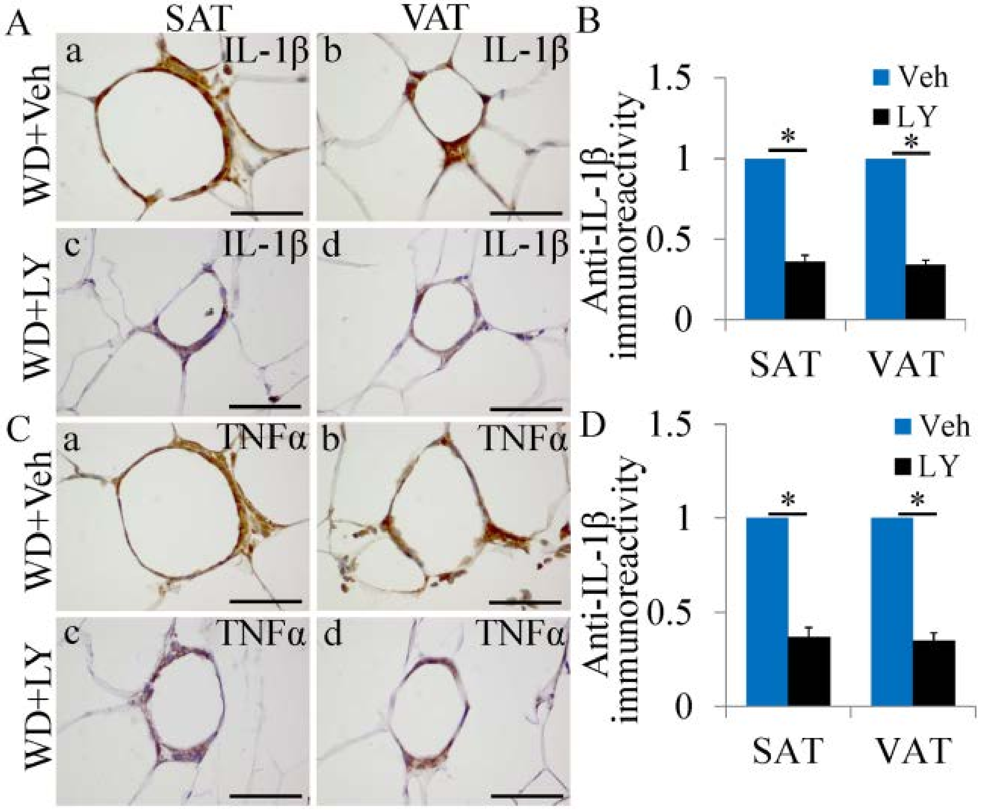

A
SAT
VAT
a
IL-1β
b
IL-1β
WD+Veh
c
IL-1β
d
IL-1β
WD+LY
B
1.5
1
0.5
0
Anti-IL-1β immunoreactivity
Veh
LY
*
*
SAT
VAT
C
a
TNFα
b
TNFα
WD+Veh
c
TNFα
d
TNFα
WD+LY
D
1.5
1
0.5
0
Anti-IL-1β immunoreactivity
Veh
LY
*
*
SAT
VAT

Figure s7

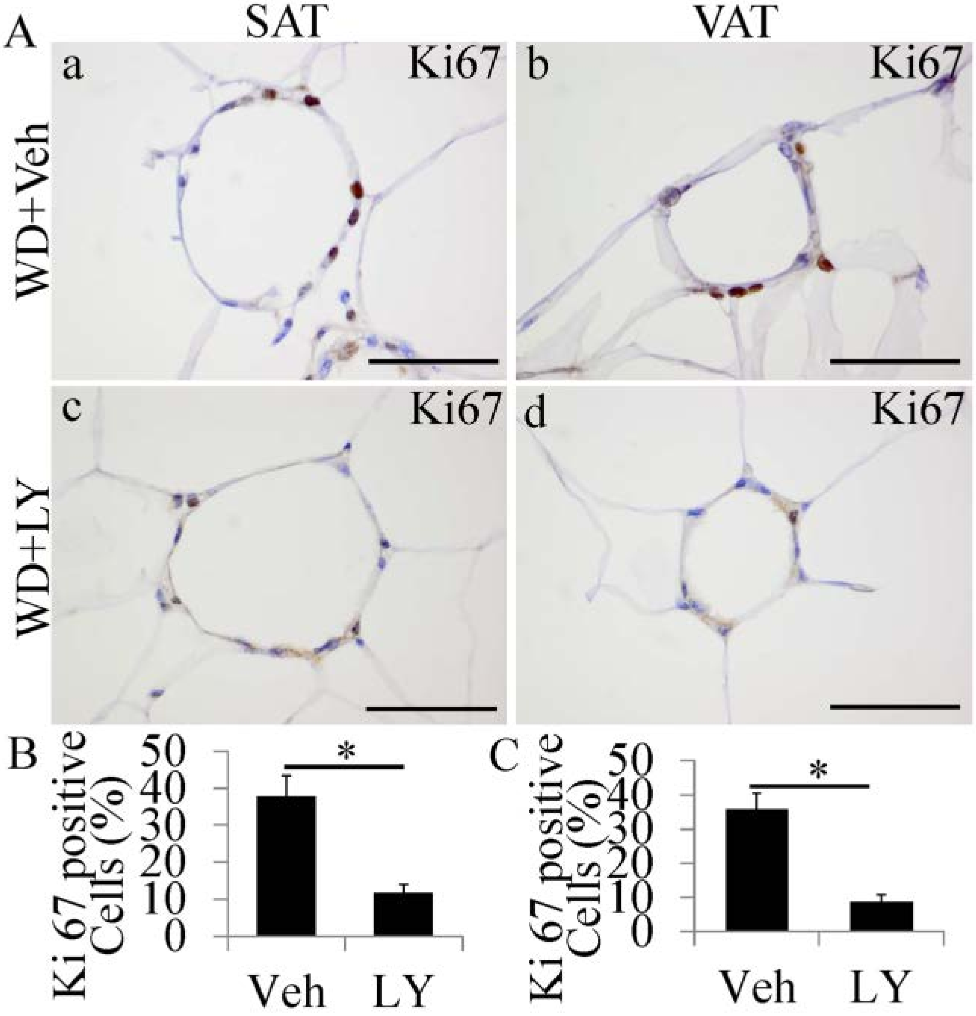
A
SAT
VAT
a
Ki67
b
Ki67
c
Ki67
d
Ki67
WD+Veh
WD+LY
B
Ki 67 positive Cells (%)
50
40
30
20
10
0
*
Veh
LY
C
Ki 67 positive Cells (%)
50
40
30
20
10
0
*
Veh
LY

Figure s8

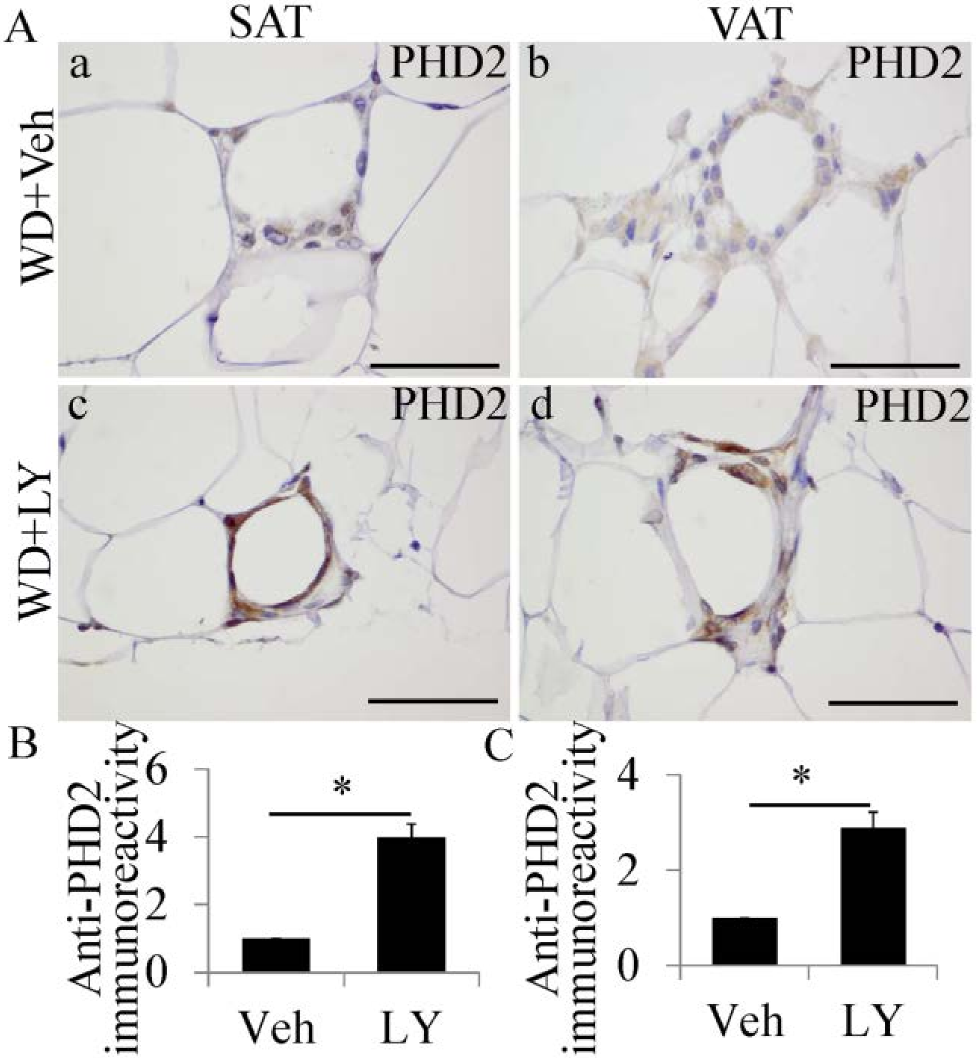
A
SAT
VAT
a
PHD2
b
PHD2
WD+Veh
c
PHD2
d
PHD2
WD+LY
B
Anti-PHD2 immunoreactivity
6
4
2
0
*
Veh
LY
C
Anti-PHD2 immunoreactivity
4
2
0
*
Veh
LY
